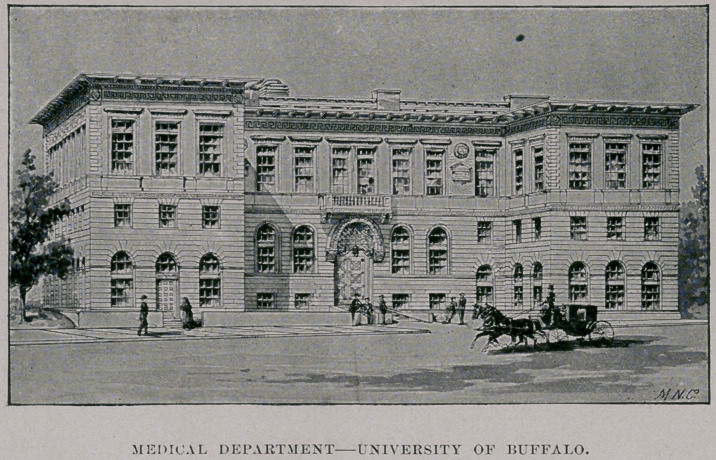# University of Buffalo

**Published:** 1893-04

**Authors:** 


### Buffalo Medical and Surgical Journal

Vol. XXXII. APRIL, 1893. No. 9.

### UNIVERSITY OF BUFFALO.

### DEDICATION AND OPENING OF THE NEW BUILDING FOR THE MEDICAL DEPARTMENT, MONDAY, MARCH 7, 1893.

Introductory remarks by Hon. James O. Putnam, vice-chancellor.

*Ladies and Gentlemen:*

The keynote of this occasion is congratulation. While congratulations are familiar to us, on the onward march of our city to wealth and greatness, it is long since the University of Buffalo, until now, has been made happy by any outward sign of material progress.

I well remember the time when, in 1846, I had the honor to be associated with several gentlemen to organize the University of Buffalo, that a medical department might take proper form under its charter. Then followed the erection of the building at the corner of Main and Virginia streets. Though not, perhaps, a thing of beauty, it served well its purpose until the needs of the college had outgrown its capacity and appointments. What should be done? Should the college creep along in the old ways far in the rear of the development of the city, or should it keep pace with the time, meet the necessities of its position, and seek a new and adequate home worthy of itself and worthy of Buffalo? There could be but one answer. The council of the university trusted the intelligent appreciation of our public on whose sympathy and aid it felt it had a right to rely. The result is before us: this classic edifice, ample in dimension, beautiful for situation, tasteful and rich in architecture, reflecting the highest credit on the architect, Mr. George Cary, and surpassing in its adaptation to its uses every other medical college of the country. Am I not right in saying congratulations are in order? To the university on this splendid result, to Buffalo on this new proof that, amid the marts of trade and commerce, science may have a seat, and go hand in hand with those imperial factors? Let us not undervalue the relation of a well-equipped medical college to the public. Science has no field of operations so closely related to human well-being as the domain of the medical profession.

I know her triumphs in other realms are the wonders of our time. She has put a girdle round the globe for the instantaneous transmission of thought. She has harnessed the "sightless couriers" of the air for man's use, and in manifold ways has multiplied a thousandfold the possibilities of human achievement. But these inventions pale in their importance as social factors before the triumphs of science in medicine and surgery, by which not only has the average of human life been increased, but the ills which flesh is heir to, have been so controlled as to vastly add to the sum of human happiness. Plagues have been stayed which once desolated nations. The causes of disease and their remedies, which for 6,000 years baffled the scrutiny of the wisest, have been discovered. Even the carnage of the battle-field has lost some of its horrors. Contrasting the hospital service of the later with the earlier wars of the century, we pay our grateful homage to the science of surgery.

The medical college is the training school which is making our life more worth living, and in country, and in city, in our private homes, and in local publics, revealing to us the laws of health which the public authorities must enforce.

These considerations have induced the liberal endowment of medical colleges by individual wealth in some of our sister cities. Not to speak of other instances, one woman in Baltimore has recently given $350,000 to build and equip the Woman's Medical College of Johns Hopkins University.

I will add that a committee for that university recently visited this building and secured copies of its plans for their own guidance. Like our library building it is already a celebrity. While Buffalo rejoices in our material enterprises, she will generously support those institutions which save her from the mean fame that follows neglect of the fair humanities.

I now declare this building opened for the uses for which it was erected.

You are awaiting the address of the evening, by one to whose energy, zeal, and courage is mainly due the success of this enterprise. Professor Doctor Cary needs no formal introduction. I have great pleasure in giving way to him.

### THE HISTORY AND PRESENT STANDING OF THE UNIVERSITY OF BUFFALO.

By CHARLES CARY, M. D., Buffalo, N. Y.

*Mr. Vice-Chancellor, Ladies and Gentlemen* :

I congratulate the University and the good people of Buffalo, and all friends of liberal education, upon the completion of this handsome and well-equipped building, which tonight we formally dedicate to the purposes of science and of instruction in medicine. For those of us who have had the matter in our immediate charge, and have been interested in establishing ourselves in the different laboratories and offices, this is a moment of supreme joy, and of pride, not unmixed with emotional excitement. The requirements in medical education, through the growing demand for practical experience in pathology, chemistry, and clinical work, have so advanced within recent years, that our new quarters, constructed expressly to meet such demands, are coming to be regarded as the most perfect building of the kind in this country, and perhaps in the world.

This work, which was undertaken about two years ago, has been pushed with zeal and perseverance, and is now a thing accomplished. Some of us have thought that our undertaking was great, but it withers into insignificance when compared with the achievement of those who first created and then built up the University of Buffalo, from 1846 to 1848. The old medical college building, which we are leaving, was certainly a credit to the time and to those by whose energies it was erected. It has answered all demands until recent years, when the development of our university and the requirements of medical education outgrew its capacities. With our enthusiasm at this moment, there is mingled a feeling of regret that the old stand must be abandoned. It has been for years associated with our arduous labors. It has been one of the landmarks of the city. It seems fitting that the history of the university should form the theme of this address, and should lead up to such suggestions as are proper to be presented with reference to its present condition and the prospects of its future.

The birth of the University of Buffalo occurred in a small office on Main street, in which were gathered some energetic young lawyers and doctors. It was there that the plan was quietly formulated to procure a charter from the legislature, and to lay the foundations broad and deep for an educational institution, with special reference to the training of doctors and lawyers, but having the powers of a university and unlimited capacities for future development.

It should be remembered that Buffalo at that time had a population not more than one-tenth as large as at present. The physicians of the entire city did not equal in number those who are today directly connected with the university. The lawyers were comparatively few, and with slender purses. The town was full of vigorous, able men, whose chief resource, however, was energy ; while the undeveloped condition of the city made the concentration of any considerable amount of money a thing almost unattainable.

The efforts of these men resulted in drafting a charter or special act of incorporation, which was passed by the legislature on May 11, 1846 (Chapter 193, Laws of 1846). It was chiefly through the energies of Nathan K. Hall, at that time representative in assembly, and of the senator from this district, Dr. Carlos Emmons, a much esteemed and eminent member of the medical profession, that this charter was procured. We can learn from old documents that it required some skillful management on the part of Mr. Hall, to overcome the opposition of a select number of our own citizens. But the aims of the broad-minded promoters of the plan were completely successful. Their charter was hewn out upon lines which provided amply for all possibilities then foreseen, "for the purpose," as is stated in the first section, "of promoting literature and science by establishing and maintaining a university." It has only been found necessary to amend it twice,—in 1847, (Chapter 34,) by adding some details relating to a quorum of the council, and to diplomas ; and in 1859, (Chapter 117,) by providing expressly for the creation of an academical or preparatory school, and for a mechanical school, and, further, for the acquisition and use of certain real and personal property and the exemption of certain real property from liability for debts.

The original charter declared that the university should have capital stock not exceeding $100,000 in amount, in shares of $20 each, and that, at least, $20,000 should be subscribed, and $2,000 paid in, before the corporation could be organized. The stockholders thus created, that is, the persons who by their subscriptions made the university possible, were to elect a "council" of sixteen members, who with the mayor and recorder of the city as *ex-officio* members, and representative members appointed by the several faculties of the university, should be the governing body of the corporation, should select the several faculties, and exercise all the corporate powers. This privilege of electing members of the council, and of filling four vacancies at the annual election, was the only one given to the stockholders. There was no suggestion, and obviously no possibility, of a dividend on their stock, or of any pecuniary profit from it. But, recognizing the possibility that the stockholders might not attend the annual meetings for such elections, the charter wisely provided that in this event the council could "supply all vacancies remaining in the board." In other words, although based upon this plan of issuing shares of stock to the original subscribers, the corporation is not a stock company in the ordinary sense of the word, but is purely an educational institution, what the lawyers call an eleemosynary corporation, which was expected to be controlled by the original donors of its funds, and by their successors and assigns, through their ownership of its stock, but to be managed for the public good and not for private gain.

After the charter was obtained, it was necessary to get subscriptions for $20,000, upon which at least ten per cent. must be paid, in order to place the corporation upon a legal footing. This was finally accomplished.

The steps taken toward organizing the university under its new charter are preserved in a small book of record, written by Nathan K. Hall, the first date in which is May 29, 1846. It is recorded that "pursuant to the act to incorporate the University of Buffalo, the undersigned have this day met at the office of Hall & Bowen, . . . and have organized by the appointment of Gaius B. Rich as chairman, and Nathan K. Hall as secretary. After which it was, on motion of Mr. J. G. Masten,

*Resolved,* That the book for subscriptions to the capital stock of the corporation created by the act to incorporate the University of Buffalo, be opened at the office of Gaius B. Rich, in the city of Buffalo, on the 5th day of June, 1846, at 10 o'clock A. M., and to remain open at the same place until the election of the council of the university, and that notice of the same be published in the city papers.

The contributions to the stock which are recorded in this book amount to 1,009 shares, or a little over $20,000. There were nineeen subscriptions for one share each, one for five shares by S. G. Austin, and three for ten shares each, by Horace M. Congar, Wm. Tweedy, and George N. Burwell. All of these were for the medical department, except one share. One subscription for 150 shares was made by Theodotus Burwell for the law library. Dr. Austin Flint subscribed for 208 shares for the medical department, and 195 shares for the medical library, and Dr. Frank H. Hamilton subscribed for 207 shares for the medical department and 195 for the medical library.

The persons who thus subscribed for the stock of the university, elected its first council, since which time the stockholders have never held a meeting or taken any further action. The first council consisted of Ira A. Blossom, Millard Fillmore, Isaac D. Sherman, Elbridge G. Spaulding, Theodotus Burwell, George R. Babcock, James O. Putnam, Hiram A. Tucker, Gaius B. Rich, Orson Phelps, William A. Bird, Orsamus H. Marshall, Thomas M. Foote, George W. Clinton, John D. Shepard, and Joseph G. Masten. Truly a distinguished list of names in the history of Buffalo. The council so chosen has continued to perpetuate itself down to the present time.

By this council, the first faculty of the medical department was immediately appointed. It was composed of James Hadley, professor of chemistry and pharmacy ; Charles B. Coventry, professor of physiology and medical jurisprudence ; James Webster, professor of special and general anatomy ; Charles A. Lee, professor of materia-medica and pathology; Frank H. Hamilton, professor of practice and of clinical surgery ; James P. White, professor ef obstetrics and diseases of women ; Austin Flint, professor of the principles and practice of medicine and of clinical medicine. These are the men who had been most active in the movement to organize the university from the outset, and upon whose subsequent labors its prosperity depended. They thus formed the nucleus of the new university.

The names and the achievements in medicine of some of these men, individually and as a body of co-laborers in building up this school of medicine, are familiar to our ears as household words, as are the names and the deeds of four others who later took places among the teachers of the school. I refer to Moore, Dalton, Miner, and Rochester, who, succeeding Hamilton, Flint, and Coventry, formed a body of medical practitioners and instructors which it would be hard to equal at that or any other day.

Let me stop for a moment to remind you that at least three of these distinguished men first made in Buffalo the reputations which led to their being called elsewhere, to fields of larger and more conspicuous labor. It is an open secret that it was the danger of a repetition of this severe blood-letting process in 1891, which convinced us of the necessity for providing ourselves with larger and better quarters for our work, so as to make it more attractive and profitable, and to enable us to keep some of our best instructors.

The first circular of the medical department, which comprised all that there was of the university, was issued in the autumn of 1846. Funds, to a considerable amount, were expended for the purchase of pathological and anatomical material, and for refitting a building on the north-east corner of Seneca and Washington streets, known as the old post-office building. The first course of lectures was begun in the spring of 1847, and at the end of four months terminated with the graduation of eighteen students, out of a class of sixty-seven. They received their degrees from Millard Fillmore, who was the first chancellor of the university, and held this position until his death in 1874.

In reviewing the requirements for graduation which were adopted in these early days, we note that they were not second to those of any other medical school in the country, and that in some respects the new university took the lead. I am convinced from the perusal of early reports that the University of Buffalo was among the first, if not the first, to adopt the requirement of practical anatomy or dissection as one of the prerequisites for graduation; also that it was one of the earliest institutions to extend its sessions to six months. But the latter step resulted in such a falling off of its students that the faculty was forced to the conviction that the profession would not uphold them in so radical an advance.

In respect to its term of instruction, and to the number, as well as the ability of its faculty, the University of Buffalo compared favorably with other leading medical colleges. It had seven professors and a term of four months, with a preliminary term of one month. The medical department at Harvard, at that time, had a faculty of seven professors, and a term of four months; Yale, six professors, and sixteen weeks; the University of the City of New York, six professors, and four months; the University of Pennsylvania, seven professors, and five and a half months.

The second session of the medical department was attended by ninety-three students, of whom thirty-four graduated. The third session was conducted through the winter, and was the term of six months already referred to. It closed with a graduating class of only nineteen, a material reduction as compared with the previous year.

At the opening of the fourth session, there was great rejoicing over the completion of what was regarded as a perfect building for medical instruction, the same one which has been occupied by the medical department from 1849 until 1893. It had become clear that such a structure was needed to insure the permanency, and to provide for the prospective wants of the institution. For this end a subscription was opened in 1847, and after a long struggle the requisite sum was obtained to secure the erection of the desired college building. It was a moment of triumph when this edifice, the complete fulfillment of the ideals of that day, was dedicated in 1849.

The subscriptions which were made for this purpose amounted to about $12,000. I cannot review the list in detail, but without invidiousness I may name A. D. Patchin, who gave $500 and was the first to be enrolled; and Jesse Ketchum, whose interest was always with our educational institutions, bore out his character by giving $600, the largest subscription. The other gifts were of sums ranging from $200 to $20.

An engrossed copy of this subscription to the building fund signed by the original donors, was for many years in the possession of Orsamus H. Marshall, who was a member of the first council of the university, and was its second chancellor, and always its firm and devoted friend. This copy is, tonight, to be presented to the university by his son, Charles D. Marshall, and will be preserved among our most valued records of that early period of storm and stress. I may add, here, that the generosity of those whose subscriptions have aided and shall aid in our latest development, will be recorded and preserved in the same way.

At the dedication of the building, Dr. Austin Flint delivered the principal address, in which he described its features and advantages. Many of the historical facts which I am able to present to you tonight are taken from his paper. He describes the discouragements of the original subscription committee, consisting of Fillmore, Hall, and Marshall, and gives great praise to Orlando Allen, who came to the aid of the movement at a critical point, and by his labors contributed largely to its success, and to James Hollister, and in general to the younger men of the city. It was to these young men of energy but of limited means, rather than to the larger capitalists of the day, that the credit for its final triumph was chiefly due.

Dr. Flint spoke of the new building, the pride of their hearts, as follows :

The building is situated on the corner of Virginia and Main streets, in a pleasant part of the city, somewhat elevated and sufficiently removed from the center of business to be free from the noise and disturbance of the latter. The style of architecture is Norman, or more properly Romanesque, a style regarded as peculiarly appropriate for buildings designed for similar purposes. The windows are arched ; the walls of red sandstone, resembling the New Jersey redstone, of which Trinity Church, of New York, is built. They present a rough, unhewn surface, the effect of which is to give an appearance of massiveness, age, and strength to the building, and which accords with the style of architecture adopted. The lecture rooms present one peculiar feature which has excited great attention. They are seated with cast-iron chairs, with bottoms firmly screwed on *cushioned benches.* They are pleasing to the eye and exceedingly comfortable to the sitter, in fact combining all the advantages of a luxurious arm chair. The right arm of each chair is expanded for the purpose of taking notes, and, the whole being painted, it presents quite an elegant appearance. We know of no educational institution in which similar provision for the physical ease of the pupil exists. If the faculties of the mind are inactive, it will not be because the attention is absorbed by uneasy sensations of the body ; and while the latter are obviated, the indulgence of postures favorable to indolence and sleep is effectually prevented.

The advantages of the medical school, in its new location, were greatly increased by the establishment, in 1848, of a hospital near it, on Virginia street, under the direction of the Sisters of Charity, with a capacity of 100 beds, which was soon increased to 200.

It would be interesting to follow in detail the history of the university, or rather of the medical department, which was its only outward manifestation, from its beginning to the present time ; but suffice it to say, that the council and medical faculty, having succeeded through gigantic efforts in carrying out their immediate intention, rested. No further outward developments followed until 1886.

In the meantime, the name and the idea of the broadly-planned University of Buffalo passed out of the public view, and were almost forgotten. The Medical College, as its one existing department was commonly called, was well known. Its professors were respected as teachers and as leaders in their profession. Its building, grown dark and weather-stained, frowned with its grim portal upon what had become a busy and crowded part of Main street, and sent forth classes of long-haired and be-shawled students, whose supposed practices were such that they and their place of instruction were rather feared and avoided by the masses of people. But all the far-reaching possibilities of a great uni versity a seat of general education, which were locked up in the terms of our charter, and even, as I have said, the very name of the university, were forgotten.

It is not strange, then, that a prominent and well-informed citizen, when recently asked for a subscription to the University of Buffalo, for its new medical building, said: "What is the University of Buffalo? Is it a new thing? I never heard of it;" and could hardly be persuaded of the facts. Be it remembered, however, for the encouragement of future efforts, that this same gentleman, when the facts were explained to him, gave liberally to help us in our present needs. And this points a moral, which can be drawn from the earlier and later struggles of our institution—that the people of Buffalo are liberal with money and with aid of every kind to assist all worthy enterprises which are fully, and candidly, and earnestly presented to them. So long as we deserve their support and make them understand our needs, they will not fail us.

The records of the council meetings, between 1850 and 1886, contain an occasional suggestion for the establishment of a law department; and, curiously enough, the medical journals contain memoranda of suits for malpractice, and for libel, which naturally diverted the energies of the then young faculty from any notable attempt at enlarging the scope of the institution.

But, during these early years, Flint, Dalton, and Hamilton gave themselves up to the occupation of teaching and of recording their clinical cases, and to preparing for their subsequent literary labors, which are now embodied in volumes of the very highest authority in their several fields of medicine, physiology and surgery. Dr. White, although not distinguished as an author, became an authority of the first rank in his special department, obstetrics. He assumed control, and was practically the manager of the infant university, from the time when he aided at its birth in 1846 until his own death in 1881, and held the reins of government over it with a firm and judicious hand. It is from the time of his death that the regime of the present faculty practically begins.

The medical department had the usual ups and downs of a poor and struggling technical school, but in the main it grew and progressed, as it could not fail to do with such a body of instructors.

All of these early teachers have passed away, except noble old Dr. Moore, who still preserves a connection with the school—a living example of the greatness of the men who have been connected with this college. Their places have been filled by younger men, who have labored hard, and, I venture to say, successfully, to keep up and constantly to advance the standard of practice and of medical teaching which their predecessors had set so high.

It must be left for others than myself to make further comments upon the recent work of the medical department and its instructors. Certain it is that the school has grown under their management in numbers, and influence, and public respect. The present governing faculty still consists of seven members, who, named in the order of seniority, are Charles Cary, Matthew D. Mann, Roswell Park, Julius Pohlman, Charles G. Stockton, John Parmenter, and Herbert A. Hill ; but with them is associated a large body of professors and instructors in special subjects, which brings the number of the teaching force up to forty-four. Its body of students has advanced to 157. Its course now consists of three years, occupying over seven months in each year, besides a spring term of two months. It is now, as it was in earlier days, one of the leading schools of medicine in the country. The city of Buffalo has a right to be proud of it ; and we, its teachers, felt that we had a right to ask the people of Buffalo to help us to provide ourselves with new and adequate quarters, such as we are now to occupy.

In 1886, the medical faculty urged the council of the university to establish a department of pharmacy, in which students could attend jointly some of the lectures of the medical faculty, in addition to courses of instruction from a special faculty in pharmacy, and could receive the degree of graduate in pharmacy. This department was then created. Its first faculty was composed of the following able teachers : R. A. Witthaus, professor of pharmaceutical chemistry ; E. V. Stoddard, professor of materia medica ; Willis G. Gregory, professor of pharmacy ; David Kellicott, professor of botany ; F. P. Vandenburgh, professor of general and inorganic chemistry. It is due to Dr. Vandenburgh to say that this department was organized largely through his untiring energy.

From its first establishment the department of pharmacy met with a success far transcending the most hopeful expectations pictured at the time of its creation. Its first year opened with a registration of thirty-nine students, who showed keen appreciation of the opportunities offered to them; and there has been an increase in attendance in this department, until this year the class numbers sixty-five. At no time has its onward march met with a single check.

No additional departments were organized until May, 1891. Then the law department was created, with Judge Charles Daniels as its dean. It must, however, be recalled, that the Buffalo Law School had been in existence for several years prior to that date, having been founded in 1887 by members of the local judiciary and bar. It was this existing law school, with its body of instructors and students, which united with us and became the law department of the university. Its first faculty was as follows: Charles Daniels, dean and professor of constitutional law; LeRoy Parker, vice-dean and professor of the law of contracts and municipal law; Charles Beckwith, professor of equity jurisprudence; George S. Wardwell, professor of the law of torts; Albion W. Tourgee, professor of legal ethics; Spencer Clinton, professor of the law of property; James Frazer Gluck, professor of the law of corporations; George Clinton, professor of maritime law and admiralty; John G. Milburn, professor of the theory of law, codes, and codification; Adelbert Moot, professor of the law of evidence; Tracy C. Becker, professor of criminal law and procedure, and medical jurisprudence; Charles P. Norton, registrar and professor of the law and practice of civil actions; Carl T. Chester, professor of the laws of marriage and divorce, and special proceedings; E. Corning Townsend, secretary and treasurer, and professor of the law of domestic relations.

Encouraged by the success which they had met in establishing these departments, the council, in 1892, with the coöperation of some of the most competent dentists of the city, organized a dental department. The department had a governing faculty of four professors: William C. Barrett, professor of the practice of dentistry and dental pathology; Alfred P. Southwick, clinical professor of operative dentistry; Herbert A. Birdsall, professor of dental materia medica and therapeutics; Franklin E. Howard, professor of operative dentistry. This original faculty has associated with themselves sixteen other professors and instructors in special subjects. The department brought together forty-seven students in its first year, which is more than any other school of dentistry is known to have had at its opening session.

The total number of persons now connected with all departments of the university, as governors, teachers, and students, is 483. So large a body of earnest workers in the cause of education cannot fail to exert an influence for good in the community, which will be more and more felt in the future.

The establishment of these three new departments within the past few years and the conspicuous progress of the medical department, have awakened the people of Buffalo to the existence of a university in our midst, in fact as well as in name. But the end is not yet; the developments which should make the university fully worthy of the city are only fairly begun. We shall call upon you to help in carrying them forward.

It seems proper at this time to make public the proprietorship of this institution. Enough has already been said to explain the basis of the organization of the university, upon the theory that the original subscribers to its funds were to become stockholders, and, as such, were to elect the council to manage its affairs. But, as already stated, after electing the first council, the subscribers to the stock never held another meeting or exercised any further powers. No certificates of stock were issued to them, and apparently none were expected, for, as Dr. Flint states, "the public spirit exhibited by these subscribers is enhanced by the fact that they gave freely, without expectation of pecuniary return." The affairs of the corporation, while it consisted only of a single department, were thus managed nominally by the council, but actually by the medical faculty, through the acquiescence of the council.

It has been supposed by some who chose to withhold their aid from the university that it was controlled by one person. But in all fairness to any one who may have been suspected of such business acumen, it should be stated that at no time in its history, until now, has there been any documentary evidence that there were stockholders. Within recent years the looseness of the existing state of things, with no stockholders to exercise the powers conferred by the charter, was for the first time appreciated, and an effort was made to correct this irregularity and defect of the early proceedings. Orsamus H. Marshall, as chancellor, was the first to attempt to place the university upon an unassailable footing, by procuring assignments of the original stock subscriptions, with the idea that the stock should then for the first time be issued; but before his work was completed, death withdrew him from the council. In 1884, the attempt was resumed. Assignments of their original stock subscriptions were procured from some of the subscribers, or their representatives. These assignments were made to the then members of the council, to be held by them as joint tenants for the benefit of the university.

Our new building, however, has effected a more complete transfer of all the stock subscriptions than could probably have been accomplished under any other circumstances than an urgent demand for funds. It was necessary to borrow $100,000 by a mortgage upon the property. The law required that before this could be done, the consent of at least two-thirds of the stockholders should be obtained. To satisfy this demand it was necessary that there should be stockholders. In the face of such an emergency, the work was resumed with renewed ardor, in 1892, by a special committee appointed by the council, of which Ansley Wilcox was the chairman. By corresponding with heirs and by seeking them out, there was finally procured for the council of the university the assignment of 809 shares,—more than four-fifths of all the original stock list, including the large subscriptions made by Drs. Austin Flint and Frank H. Hamilton. The stock was then, for the first time, issued to members of the council as joint tenants, to hold it for the benefit of the corporation. The readiness with which all who were approached yielded their claims and made the required assignments to the council, has enabled the University of Buffalo to be placed upon an absolutely firm and permanent legal footing, and has hastened the completion of the new building which we are occupying tonight.

It remains for us to consider more in detail the necessities which led to the erection of our new medical college building, and what has been achieved in this connection, and what is to be done.

It was for many years manifest to those actively connected with teaching in the university that the quarters of the medical department, on Virginia and Main streets, had been outgrown. Plans were prepared and submitted to the council for a very material addition to the old building. The matter was considered in all its phases, and much preliminary work was done. But the first formal record of any action is that taken on May 6, 1891, when the die was cast, and it was resolved to complete the purchase of land on High street, part of the Goodrich lot; and, further, that a committee of three be appointed from the council to form, with two members from the faculty, a joint committee, whose duty it was to procure plans for a complete building for the medical department, at a cost not to exceed $125,000. At that time a considerable amount of money had already been subscribed by some of our public-spirited citizens, and the possibility of effecting this purchase and the erection of a new building was apparent. The lot was purchased for $22,275.

That the medical department might not be embarrassed by any debt remaining upon the new building, the council passed a resolution to the effect that it was not for the best interest of the university that any portion of its current income be diverted from the channels of teaching, and that whatever indebtedness might be incurred in the present undertaking, be provided for by the council, until paid by the mortgage and sale of property, and by subscription.

The joint committee of the council and faculty consisted of Charles Cary, chairman, Roswell Park, Edmund Hayes, J. J. Albright, and Robert Keating, who procured the service of George Cary as architect. In November, 1891, a set of plans was submitted for approval. Bids were received, and the same committee was authorized to contract for the erection of the new edifice at a cost of $124,888. From that time the building has been carried forward rapidly to completion. The first lecture was delivered in Alumni Hall, on January 6, 1893, by the present speaker. Since that date we have gradually been moving into it.

It is not too much to say that an unusual share of the credit for this building is due to our architect. From the first completion of his plans, which combined artistic merit with a practical adaptation to the necessities of the structure and the requirements of its occupants, on to the reception of the contractors' bids, which furnished a rare pleasure by coming within the estimates, and finally down to the completion of the whole in a substantial and durable manner, within the original estimate, the architect's work has given thorough satisfaction to the building committee. It is but just to him to award this meed of praise.

I should not properly follow the example of my distinguished prototype, Dr. Flint, on the similar occasion in 1849, to which I have referred, if I failed to make formal mention of some of the many beauties and advantages of our new building. Your inspection of it makes it unnecessary for me to enter into much detail. And as you are not sitting on the luxurious cast-iron chairs, with "cushioned benches," which Dr. Flint immortalized, (and which are still preserved in our lecture hall, and also in the dispensary, for the benefit and delight of future generations of students,) I am warned to be brief.

But to preserve for posterity, as Dr. Flint did, our present impressions of our new home, let me say that the building is classic in its architecture. Both the architect and the committee have had before them the idea, which they have tried to realize, that in its exterior design and in all of its internal appointments and ornamentation, there should be a purity and simplicity of style and of construction, combined with usefulness and economy both of space and material, and a direct and obvious adaptation of all parts to their known purposes, which would make it a structure tending directly to the elevation and refinement of our youth, and in itself an educating influence. Therefore it is that the faces of Flint and of Hamilton are placed in relief upon its facade. And in the main college hall you may see, emblazoned in letters of gold, the names of eminent physicians of foreign countries, and of the United States, and of some natives who are given a place in this grand company on account of their connection with the University of Buffalo.

It was partly for the sake of this educating influence, as well as for economy, strength, safety, and cleanliness, that the open system of internal construction was adopted, which avoids all concealed spaces, and exposes every plank and timber, and every pipe to view,—so that a walk through the building may be said to be a lesson in anatomy.

The building contains three lecture rooms, with a seating capacity varying from 100 to 350 chairs; several private offices; a dispensary large enough to care for 250 patients daily; chemical-anatomical, physiological, pathological, and bacteriological labora, tories, and excellent accommodations for the medical and scientific library, as well as rooms devoted to the use of the various branches of the medical department, and some allied purposes. The library rooms are fire-proof, and contain our present library of about 4,000 volumes and 5,000 unbound pamphlets. They are capable of holding about 40,000 volumes.

To meet the cost of the new lot and building, amounting to about $150,000, we had our old college property, which is estimated to be worth $70,000, and which we have mortgaged, with the new property, for $100,000; and we have raised by subscription $45,000. With these limited resources, trusting in providence and in the liberal support of the people of Buffalo, we have carried through our undertaking. But this statement shows that we are left with a heavy debt and a large interest charge to be met.

The university began at once to feel the new position which it was to hold in the community. Ere the building had been much more than started, a liberal bequest of $20,000 was received by the will of Jonathan Scoville. This sum practically reduces our debt, including accumulated interest, to $20,000, or $25,000 depending upon what we realize from our old property.

Mrs. John C. Graves presented the beautiful medallion heads of Doctors Hamilton and Flint, which she herself made, to adorn the entrance to the building.

Mrs. Esther A. Glenny gave, as a memorial to her brother, Dr. Burwell, his library of 1,418 volumes, besides pamphlets, to collecting which he had devoted many years of his life. In addition to this, Mrs. Glenny endowed the university with $2,500 for the maintenance of the Burwell library.

It should be mentioned here, as a matter of history, that our library was really started by James P. White, through the gift of the valuable medical books of his father, Dr. White.

All of us who have been interested, either as teachers or as managers of our new and enlarged enterprise, have felt that this undertaking has been the means of placing us in such relationship with the liberal people of the city, that the means will not be lacking to pay off our indebtedness, and to sustain any undertaking directed to enlarging our sphere of usefulness in education.

In this belief it is already proposed to establish a preparatory school, which is expressly provided for in our charter, as amended in 1859, where the youths of our city may receive a preliminary education for the ordinary business of life, or such as to enable them to enter the academical departments of other colleges, or the schools of any of the learned professions. This is likely to be the next step in the expansion of our scheme.

It is also urged that we should create a school of veterinary medicine, which would help to place this branch of medical science in its proper position in relation to the community and to modern medicine.

Following these, in natural order, would come a separate department or some special provision "for instruction in practical mechanical science, mining and engineering," and also, perhaps, "in the science of teaching." The express authority to establish such courses of instruction is likewise found in the charter as amended in 1859.

I know of no reason why there should not be an art school, organized and conducted under the broad charter of our university. This might come about either through the association with us of some existing school of art in Buffalo, or through the establishment of a new one. I venture to suggest, but without committing any one to such a proposition, that this would be a most desirable consummation.

These ideas, if carried out, would provide us with all of the departments of instruction which are usually included in a university scheme, except an academical department, or school of letters and liberal arts, which is ordinarily in this country the central and most important member of such institutions.

At this point our dream of developments to come must stop. It seems hardly probable that Buffalo will, at any time in the near future, be a suitable place for the establishment of such an academical department; and yet, who can tell what the future may bring forth? It is sufficient for us to know that our charter gives ample authority for the creation of such a department, if the demand for it arises and if the means to establish it are forthcoming.

Enough has been said to indicate how large are the possibilities of our future, and what an amount of work may lie before us in carrying on to its complete development the scheme of university education which was thought out by Flint, Hamilton, and White, and by Hall, Marshall, Fillmore, Ketchum, Patchin, Burwell and Allen, and their associates, in 1846. Let us devoutly hope that we shall not fall short of realizing their anticipations, or fail in the duty to our university and to the city which our relations with this body impose upon us.

---

We note with pleasure an addition to our exchange table in the form of The Buffalo Medical and Surgical Journal, which has become one of the most acceptable journals of the present day, owing to many strong features it possesses, most prominent of which is its Review Department. The genial editor, Dr. Potter, can well be proud of his "Reviews," for they are deserving of only the highest praise and bid fair to outrival the well-known "Reviews" of the *American Journal of the Medical Sciences*.— . . .

## Figures and Tables

**Figure f1:**